# Editorial

**Published:** 1861-03

**Authors:** 


### Editorial.

### OUR IGNORANCE

SOMETIMES discourages, and sometimes disgusts us; hence, we prefer to expose it, rather than be tortured by its continuance. So here goes. We didn't know it was very cute—there now, that's a vulgarism—we didn't know it was very *acute* to have the good sense to use iodine "in the treatment of chronic periodontitis accompanied by a constant discharge of pus," till we learned the fact from the editorial department of the February *Cosmos*. There we are told that, "useful as the remedy is, it is only, however, within the past four years that the attention of the profession was first directed to it by Dr. Garretson," which may be all true enough; but so common was this agent thus used, in these regions, when we came into the profession, that we, ignorantly it is true, supposed every *dentist* used it whenever the case seemed to require it. We used it for such cases before we came into the profession. The first time we ever saw Dr. Atkinson, if our memory is correct, he was pumping tincture of iodine into an alveolar abscess. This was in 1854. But he was, perhaps, like us, ignorant of the fact that there was need to direct the attention of the profession to it; and hence, this duty was left for Dr. Garretson, and we are heartily glad he has performed it. But we still think that a reasonably extended medical education would suggest the remedy, without special attention being called to it.

Then, there is the cork attachment to a lathe, the paternity of which seems to be in dispute. And we hope those who are settling this question will next tell us who invented the wagon. Till this cork dispute arose, we supposed every body that wanted to, used corks attached to lathes, in polishing plates. We saw Dr. Taft using them in 1849, but not attached by a "screw," or *gun-wiper* contrivance, but inserted into a cylinder, attached to the lathe. They are in common use out here.

Now, the only excuse we can plead for our ignorance is, that we came into the profession at the eleventh hour; for, though one of the *old ones*, our age was mainly attained in another profession.

W.

### DR. CHARLES BONSALL.

By the Minutes of the last meeting of the Mississippi Valley Association, it will be seen that Dr. Bonsall has resigned his office and membership in the Association, and has retired from the practice of dentistry. How strange the Minutes of the Association will look without the name of Dr. Bonsall among the "Members present!" for he was *always* present. And no "Treasurer's Report," with his familiar signature appended! We scarcely know how to record the Minutes without it. No other Treasurer has ever reported to the Association; and in all the seventeen years of his official services, the reports of the auditing committees, appointed by his request, have re-echoed the stereotyped "Your committee, having examined the Treasurer's accounts, find them correct." Dr. Bonsall was one of the founders of the Association, attended all its meetings, took an active interest in all its proceedings, and retires from it only as he retires from the profession. He has served the profession long, and he has served it well; and, as he retires from it, to engage in pursuits more congenial to his circumstances, and more compatible with his age, he carries with him the best wishes of all its members. His resignation reminds us that we must all resign our places some day—not only as members of the dental profession, but as actors in the great drama of life; let us, therefore, follow the upright example of our retiring father and friend, trusting that when called to give an account of our stewardship, it may be said of each of us, "having examined his accounts, do find them correct." W.

### IODINE.

At the suggestion of Dr. Atkinson, we have used iodine either in the form of tincture, or crystal, in the treatment of sensitive dentine, and with very marked success. In cases where there is a sensitive condition, that renders treatment necessary, apply a pledget of cotton moistened with the tincture of iodine, and seal up with wax, and let it remain for twelve to twenty-four hours, when the sensitiveness will be entirely gone, or very much relieved. By enclosing a crystal of iodine in the cavity, the same object will be attained. The dentine often becomes sensitive after excavation, if the cavity is left unfilled for a day or two; this is prevented by the application of iodine. This agent accomplishes the object better than creosote or any similar agent that we have tried. T.

